# Bibliographical

**Published:** 1874-04

**Authors:** 


### BIBLIOGRAPHICAL.

The Volume of Transactions of the Thirteenth Annual Session of the American Dental Association has just come to hand, and presents a very fine appearance—better, indeed, than any former volume; the typography, paper, etc., are superior, and reflect credit upon the Publication Committee.

The matter is good and shows progress in the profession. We think, however, it would have been better if the Committee had taken a little more liberty in selecting and trimming the matter published. The report is, perhaps, as good as can generally be obtained; but we can hardly see the propriety of publishing a paper that had been read before another Society and published in its volume of Transactions.

This Volume should be in the hands of every dentist. It can be obtained of Dr. M. S. Dean, of Chicago, Secretary of the Association. All the members of the Association who are square on the Treasurer's books are entitled to, and have each received, a copy.

